# Large-scale transcriptome changes in the process of long-term visual memory formation in the bumblebee, *Bombus terrestris*

**DOI:** 10.1038/s41598-017-18836-3

**Published:** 2018-01-11

**Authors:** Li Li, Songkun Su, Clint J. Perry, Maurice R. Elphick, Lars Chittka, Eirik Søvik

**Affiliations:** 1 School of Biological and Chemical Sciences, Queen Mary University of London, London, E1 4NS UK; 2 College of Bee Science, Fujian Agriculture and Forestry University, Fuzhou, 350002 China; 3 Institute for Advanced Study, Wallotstrasse 19 D-14193 Berlin, Germany; 4 Department of Science and Mathematics, Volda University College, 6100 Volda, Norway

## Abstract

Many genes have been implicated in mechanisms of long-term memory formation, but there is still much to be learnt about how the genome dynamically responds, transcriptionally, during memory formation. In this study, we used high-throughput sequencing to examine how transcriptome profiles change during visual memory formation in the bumblebee (*Bombus terrestris*). Expression of fifty-five genes changed immediately after bees were trained to associate reward with a single coloured chip, and the upregulated genes were predominantly genes known to be involved in signal transduction. Changes in the expression of eighty-one genes were observed four hours after learning a new colour, and the majority of these were upregulated and related to transcription and translation, which suggests that the building of new proteins may be the predominant activity four hours after training. Several of the genes identified in this study (e.g. *Rab10*, *Shank1* and *Arhgap44*) are interesting candidates for further investigation of the molecular mechanisms of long-term memory formation. Our data demonstrate the dynamic gene expression changes after associative colour learning and identify genes involved in each transcriptional wave, which will be useful for future studies of gene regulation in learning and long-term memory formation.

## Introduction

Learning and memory enable animals to modify their behaviour in response to environmental changes^1^. Understanding how animals store and retrieve memories can provide valuable information for the development of effective therapeutic treatments for memory disorders, such as Alzheimer’s disease and cognitive dysfunctions that develop with normal aging.

The storage of information in long-term memory requires transcriptional and translational regulation in the brain^2,3^. Utilization of pharmacological approaches, in vertebrates and invertebrates, has revealed that at least two waves of transcriptional activity are needed for long-term memory formation, one occurring immediately following the time of training and another occurring 3–6 hours after training^4–6^. Immediate early genes, encoding transcription factors and other DNA-binding proteins, are involved in the first transcriptional wave. The protein products of immediate-early genes act on a wider set of target genes (the second transcriptional wave) that are responsible for synaptic reorganization, which is considered the basis of learning and memory^4,7^. The functions of specific genes in learning and memory formation has been established in a variety of model organisms, such as *Drosophila*, honeybees and mouse^4,8–10^. However, our knowledge of the global gene expression changes in response to specific learning and memory is still limited.

High-throughput sequencing technology has made it possible to examine the genome-wide transcriptional response to specific behaviours, resulting in the identification of many known or novel transcripts involved in certain behaviours at high resolution and large scale. For example, using this technology, the transcriptional responses to diseases in humans^11^, to memory formation in specific learning tasks in rats^12^, and to social behaviours and specific learning in insects^13,14^ have been examined.

Bees’ remarkable cognitive abilities and small brains make them ideal models to study the molecular mechanisms of cognition^15–17^. Bees can be trained to establish associations between food and specific colours, shapes and patterns, and even to learn the concepts of sameness and difference^18^. In contrast to the extensive work on the mechanisms mediating olfactory learning, relatively little is known about the mechanisms underpinning bees’ visual learning. A few other author teams have tried to screen learning-related genes by comparing gene expressions between trained and untrained groups by high-throughput sequencing, in which bees in the trained group were collected two or three days after the first training trial when the bees had formed long-term memory^19,20^. However, since some transcriptional changes responsible for long-term memory formation are initiated during or shortly after learning^6^, some genes involved in the bees’ learning and memory formation processes might have been missed in these earlier works. Thus, the aim of our study was to determine the transcriptional changes immediately or shortly following training, and identify the genes involved in the process of bees’ visual (colour) long-term memory formation comprehensively by high-throughput sequencing.

Bees were first trained to associate one clear chip with reward in a small flight arena for two days which allowed bees to get used to the environment in the arena, the artificial chips and reward levels. On day three, three groups of bees with different colour learning were collected at different times after training (0-hour Control, 0-hour Learning and 4-hour Learning). Bees in the 0-hour Control group were trained on one clear chip and were collected immediately after training; bees in the 0-hour Learning group were trained on one yellow chip (novel colour learning) and were collected immediately after training; bees in the 4-hour Learning group were trained in the same way as the 0-hour Learning group, but were collected 4 hours after training. The 0-hour Control served as a control for the 0-hour Learning, which afforded us the opportunity to investigate genes involved in novel colour learning. Our training procedures limited the effect of the clear chip-reward association on the transcriptome changes and excluded the effects of many other factors, such as the reward level, which allowed us to be confident that the changes seen in gene expression were due to the novel yellow colour. The 4-hour Learning served as a control for the effects of time for the 0-hour Learning, i.e. this combination of groups permitted us to determine the persistence of the effects resulting from novel colour learning. Differences in gene expression patterns were expected at these two time points, i.e. we hypothesized that different sets of genes may regulate memory formation at different times shortly after learning.

In summary, we aimed at finding the specific genes involved in the process of bees’ visual long-term memory formation, which could provide future venues to work on, such as exploring how the screened genes function in the neural system and how the expression of the specific genes affects long-term memory formation. We also aimed to understand the dynamic gene expression changes shortly after associative visual learning, which could help our understanding of gene interactions that are involved in learning and memory formation.

## Results

### Retention test after colour learning

**Figure 1:**
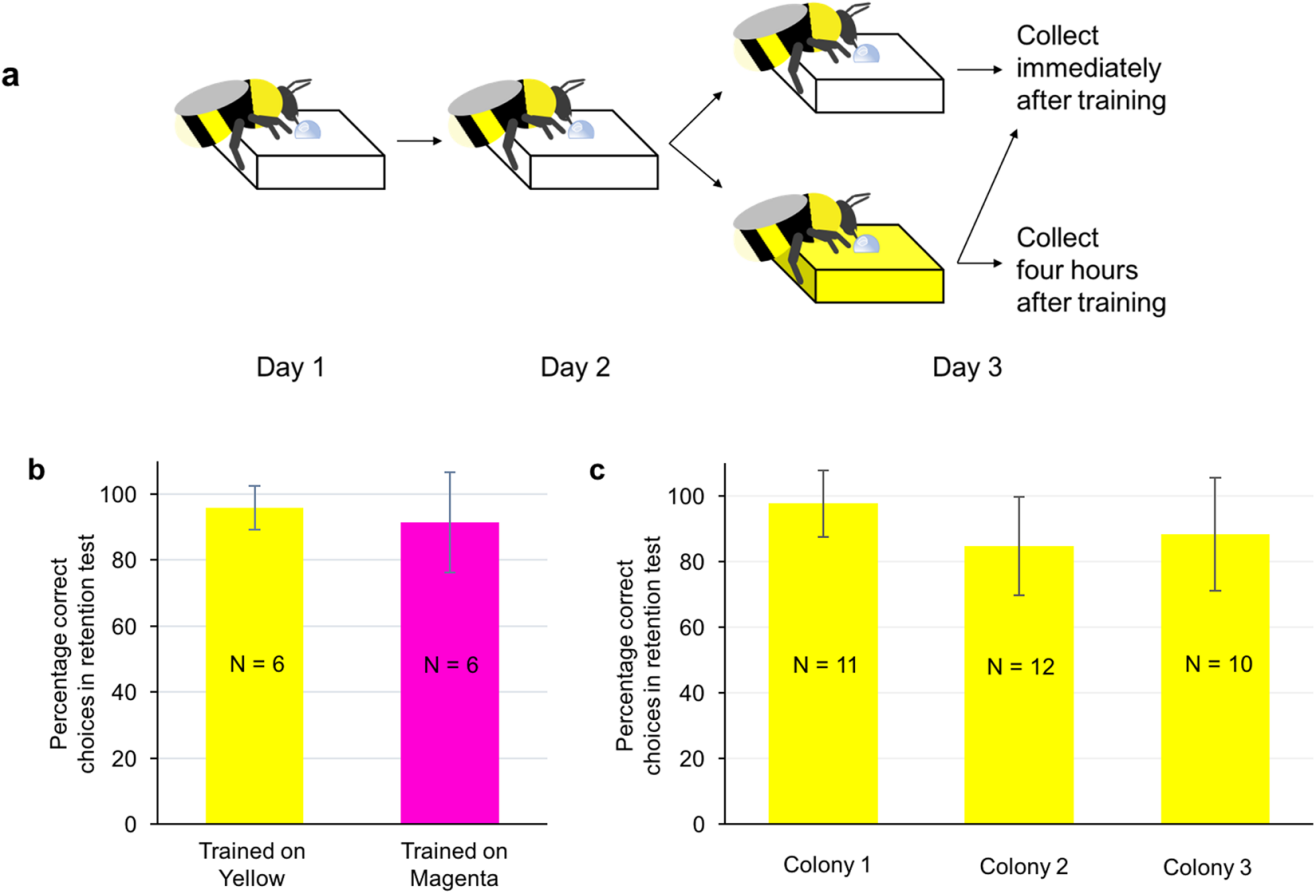
Training procedure and establishment of long-term memory with absolute conditioning. (**a**) Training procedure. Bees were trained individually to forage on one transparent chip, which contained 100 µl 40% sucrose solution (five trips with 10 min inter-trip intervals on each of two consecutive days). On day three, bees were trained to visit a chip containing 100 µl 40% sucrose solution five trips (with 10 min inter-trip interval) in one of three conditions: visiting a transparent chip and collected immediately after training (0-hour Control); visiting a yellow chip and collected immediately after training (0-hour Learning); visiting a yellow chip and kept in the hive for four hours without any further foraging experience prior to collection (4-hour Learning). (**b**) Bees form a long-term memory of a colour trained under absolute conditioning, irrespective of the colour trained. Bees discriminated the conditioned colour from a novel colour during a memory retention test (t-test, Yellow: t = 16.90, df = 5, p = 0.000; Magenta: t = 6.64, df = 5, p = 0.001, compared to chance expectation 50%). Accuracy did not differ between yellow and magenta (t-test, t = 0.65, df = 10, p = 0.532). (**c**) Bees’ memory performance did not differ across colonies. Bees in all three colonies formed long-term memory after training (t-test, Colony 1: t = 15.50, df = 10, p = 0.000; Colony 2: t = 8.05, df = 11, p = 0.000; Colony 3: t = 7.04, df = 9, p = 0.000, compared to chance expectation 50%) and bees performance on the memory retention test did not differ between the three colonies used for sequencing (one-way ANOVA, F_(2, 30)_ = 2.14, p = 0.136). The number within each bar indicates the number of bees tested. Vertical bars indicate standard deviation.

Twelve bees (one colony) was used to test whether the training procedures induced long-term memory formation. Bumblebees were trained to associate one yellow (or magenta) chip with sucrose solution for five foraging trips with 10 min inter-trip intervals. Results of a retention test conducted three days after training showed that bees remembered the trained colour with high accuracy (t-test, Yellow: t = 16.90, df = 5, p = 0.000; Magenta: t = 6.64, df = 5, p = 0.001, compared to chance expectation 50%), and the accuracy did not differ between yellow and magenta (t-test, t = 0.65, df = 10, p = 0.532), indicating that bees’ performance was not strongly influenced by an innate preference for either of the colours used in our study (Fig. 1b). Thirty-three bees (10–12 bees from each colony) were used to validate whether the bees in the three colonies used for sequencing sample collection form long-term memory after the training procedure. We found that bees in all three colonies formed long-term memory after training (the ratio of correct landings in the retention test was significantly higher than chance (50%), t-test, Colony 1: t = 15.50, df = 10, p = 0.000; Colony 2: t = 8.05, df = 11, p = 0.000; Colony 3: t = 7.04, df = 9, p = 0.000; Fig. 1c) and bees’ performance did not differ among the three colonies (one-way ANOVA, F (2, 30) = 2.14, p = 0.136).

### Alignment of sequencing reads and quality assessment

**Figure 2:**
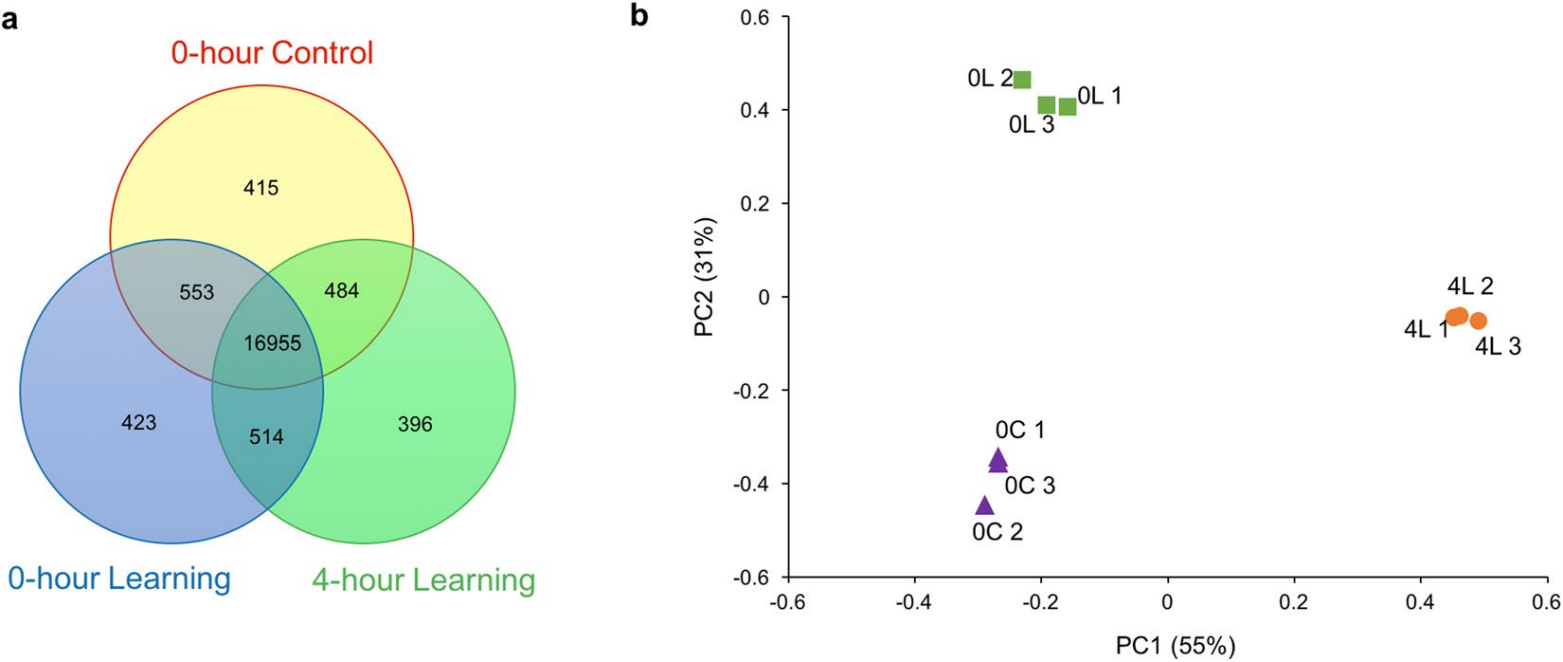
Gene expression differences associated with different learning and memory statuses. (**a**) The number of co-expressed and unique genes observed in the entire transcriptome among the three experimental groups. 86% of genes (16955) were shared among the three experimental groups. (**b**) Scatterplot of PC1 and PC2 from a principal component analysis of all samples using the gene expression values for differentially-expressed genes. The symbols represent samples from different experimental groups. PC1 and PC2 contributed 55% and 31% of the total variance, respectively. Nine samples can be separated into three experimental groups, which indicates that each learning/memory state has its own specific gene expression pattern. 0 C: 0-hour Control; 0 L: 0-hour Learning; 4 L: 4-hour Learning.

RNA sequencing yielded an average of 40.5 million cleaned reads per sample (nine samples in total), from 80.1% of raw reads (Supplementary Fig. S1). An average of 73.3% of the reads were mapped to the *Bombus terrestris* reference genome and 72.8% of the reads were uniquely aligned. An average of 66.2% of the reads were mapped to *Bombus terrestris* reference genes, of which 35.1% were uniquely aligned (Table 1). The genome uniquely mapped reads were used for later gene expression analysis. A total number of 19740 expressed genes were found in all samples, which is relatively similar to the number of genes that have been found in previous studies^21–23^; and of these, 86% were co-expressed among the three experimental groups (Fig. 2a). Quality control on cleaned data suggests high sequencing quality and equivalent data characteristics across all RNAseq samples (Supplementary Figs S2 and S3).

**Table 1:** Alignment of RNA-Seq reads to the bumblebee (*Bombus terrestris*) genome and reference genes.

Sample Name	Total Reads	Genome		Gene		Expressed Gene
		Total Mapped Reads	Unique Match	Total Mapped Reads	Unique Match	
0-hour Control 1	40722006 (100%)	31001346 (76.1%)	30828347 (75.7%)	27270084 (67.0%)	14358322 (35.3%)	16589
0-hour Control 2	40836060 (100%)	30567501 (74.9%)	30337986 (74.3%)	27671810 (67.8%)	14926440 (36.6%)	16145
0-hour Control 3	40027846 (100%)	29214232 (73.0%)	28998847 (72.5%)	26581182 (66.4%)	14119774 (35.3%)	16377
0-hour Learning 1	41025478 (100%)	30167803 (73.5%)	29984447 (73.1%)	27048580 (65.9%)	14324660 (34.9%)	16524
0-hour Learning 2	40866082 (100%)	29055426 (71.1%)	28844081 (70.6%)	26094806 (63.9%)	13860036 (33.9%)	16370
0-hour Learning 3	40670906 (100%)	29895203 (73.5%)	29670668 (73.0%)	27272970 (67.1%)	14471884 (35.6%)	16430
4-hour Learning 1	39965134 (100%)	29317201 (73.4%)	29141139 (72.9%)	26273690 (65.7%)	13822280 (34.6%)	16146
4-hour Learning 2	40133868 (100%)	29659025 (73.9%)	29445244 (73.4%)	26489752 (66.0%)	14075364 (35.1%)	16559
4-hour Learning 3	40481840 (100%)	28511125 (70.4%)	28278307 (69.9%)	26629568 (65.8%)	14036438 (34.7%)	16066

Paired-end cleaned reads (150 bp length) were mapped to the reference genome using BWA and mapped to reference genes using Bowtie.

### Unique and temporal gene expression patterns required for long-term memory formation

Gene expression was compared between the three experimental groups. A total of 110 genes were significantly different in the overall ANOVA (p < 0.01, |Fold change| ≥ 2). Information on these differentially expressed genes (DEGs) is shown in Supplementary File S1.

**Figure 3:**
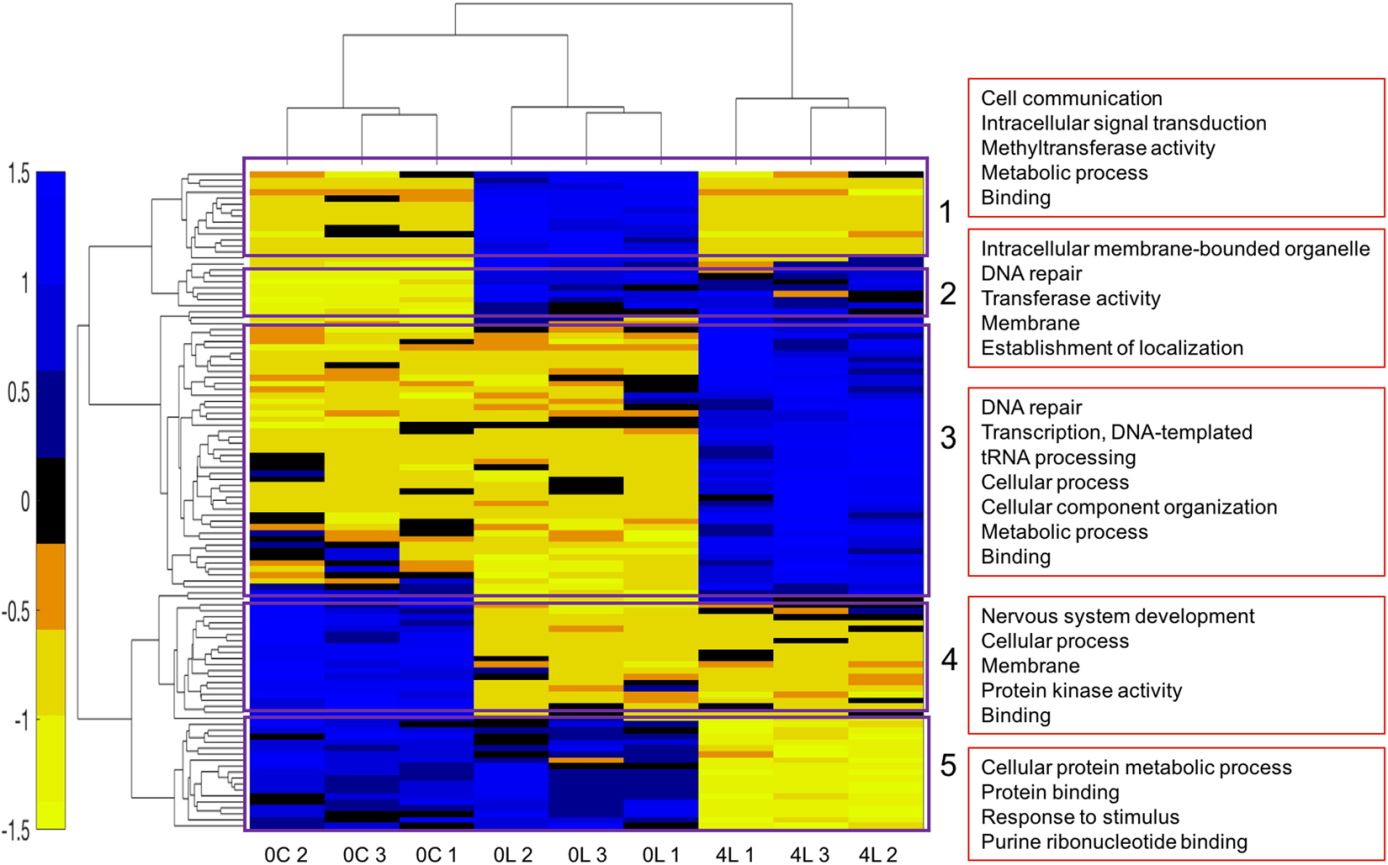
Hierarchical clustering of brain gene expression levels in bees with different learning and memory status. Each column represents a sequencing sample and each row represents a gene. Gene expression values are colour coded: blue indicates higher expression and yellow indicates lower expression. The normalized gene expression values of 110 differentially expressed genes were used for hierarchical clustering. It is evident that samples in each experimental group can be clustered together and 0-hour Control group and 0-hour Learning group had more similar gene expression patterns. In addition, several gene expression patterns were found and five of them stood out, as highlighted with purple rectangles. Red boxes on the right show the main GO terms for each cluster and the full list of GO terms in each cluster was shown in Supplementary File S3. 0 C: 0-hour Control; 0 L: 0-hour Learning; 4 L: 4-hour Learning.

PCA analysis based on the DEGs revealed that samples in the three groups were separated into three non-overlapping clusters (Fig. 2b). The differences in gene expression patterns between the three experimental groups suggest that gene expression patterns may change temporally after new colour learning. Hierarchical clustering analysis on samples also showed that the nine samples were separated by experimental treatment and the gene expression patterns in the 0-hour Control and the 0-hour Learning groups were more similar to each other than compared to the 4-hour Learning group (Fig. 3). Hierarchical clustering analysis on the DEGs showed several different gene expression patterns and five of them were of particular interest (Fig. 3; the DEGs in each cluster were shown in Supplementary File S1).

The 110 DEGs were mapped to GO terms (Supplementary File S1) which belong to one of three broad categories, Cellular component, Molecular Function and Biological Process, and 58 of them fell into the Biological process ontology (Supplementary Fig. S4). The GO terms with more than five DEGs were ‘Cellular process’ (GO:0009987), ‘Localization’ (GO:0051179), ‘Metabolic process’ (GO:0008152), ‘Response to stimulus’ (GO:0050896), ‘Single-organism process’ (GO:0044699), ‘Binding’ (GO:0005488) and ‘Catalytic activity’ (GO:0003824) (Supplementary Fig. S4). The KEGG annotations for each DEG are shown in Supplementary File S1. Seven of the top 20 enriched pathways (Supplementary File S2) were signalling pathways, including ‘Estrogen signalling pathway’ (ko04915), ‘AMPK signalling pathway’ (ko04152), ‘Dopaminergic synapse’ (ko04728), and ‘Glucagon signalling pathway’ (ko04922). Other pathways were related to transcription factors, protein synthesis (e.g. ‘Protein processing in endoplasmic reticulum’ (ko04141)) and energy generation (e.g. ‘Fatty acid metabolism’ (ko01212)). These findings suggest that the genome is highly responsive to new colour learning; genes related to signalling, transcription factors, protein synthesis and energy generation are involved in learning one novel colour and long-term memory formation.

### New colour learning triggers gene expression changes immediately after training

Compared with the 0-hour Control group, the 0-hour Learning group differentially expressed 55 genes (28 upregulated and 27 downregulated) as a result of new colour learning. Most of the upregulated genes in the 0-hour Learning group when compared with the 0-hour Control group maintained high expression for a short time (less than four hours, Cluster 1). The differentially expressed genes in Cluster 1 were found to belong to GO terms ‘Binding’ (GO:0005488), ‘Catalytic activity’ (GO:0003824), ‘Cell communication’ (GO:0007154), ‘Signalling’ (GO:0023052) and ‘Metabolic process’ (GO:0008152) (Supplementary File S3). The pathways detected for DEGs in Cluster 1 mainly fell into the Signal transduction category (such as ‘AMPK signalling pathway’ (ko04152), ‘PI3K-Akt signalling pathway’ (ko04151), ‘MAPK signalling pathway’ (ko04010)) and Nervous system category (‘Serotonergic synapse’ (ko04726), ‘Glutamatergic synapse’ (ko04724), ‘Dopaminergic synapse’ (ko04728)) (Supplementary File S4). PI3K-Akt signalling pathway and MAPK signalling pathway have both been found to be important in learning, long-term memory formation and synaptic plasticity^24–26^. The genes found to be upregulated in bumblebees after new colour learning are involved in the regulation of certain neurotransmitters (e.g. serotonin, glutamate and dopamine). These neurotransmitters have all previously been shown to play an important role in associative learning^27–29^, and our results suggest that they also play a role in colour learning in the bumblebee. Our results also showed that a group of signalling-related genes (including ras-related protein Rab-10 (*Rab10*); protein phosphatase PP2A 55 kDa regulatory subunit (*tws*); SH3 and multiple ankyrin repeat domains protein 1 (*Shank1*); dual specificity protein phosphatase 10 (*Dusp10*)) responded quickly (immediately after training) to new colour learning and their expression dropped 4 hours later.

Eight of the 28 upregulated genes kept at high expression level (compared with 0-hour Control group) and the 27 downregulated genes kept at low expression level (compared with 0-hour Control group) consistently after new colour learning (Cluster 2 and 4), which suggested on-going changes. The significantly enriched pathway in Cluster 2 was ‘Base excision repair’ (ko03410), which may indicate the high transcriptional activity shortly after learning. Half of the enriched pathways in Cluster 4 belonged to organismal systems (Supplementary File S4), most notably the endocrine system, such as ‘Insulin resistance pathway’ (ko04931) and ‘Estrogen signalling pathway’ (ko04915), suggesting that the endocrine system may become less active during learning.

### Protein synthesis-related genes activated four hours after new colour training

Comparing the 0-hour Learning group with the 4-hour Learning group, 81 DEGs (46 upregulated and 35 downregulated in 4-hour Learning group) were found. The upregulated genes in the 4-hour Learning group (Cluster 3) were enriched for genes related to ‘Metabolic process’ (GO:0008152) (e.g. ‘Nucleic acid metabolic process’ (GO:0090304), ‘Cellular macromolecule metabolic process’ (GO:0044260), ‘Organic substance metabolic process’ (GO:0071704)) and ‘Cellular process’ (GO:0009987) (Supplementary File S3). Many pathways identified in this cluster were associated with genetic information processing (replication and repair, transcription, translation) and signal transduction (e.g. ‘Hippo signalling pathway-fly’ (ko04391) and ‘TNF signalling pathway’ (ko04668)) (Supplementary File S4). These results suggest that signalling- and protein synthesis-related genes were activated several hours after new colour learning. The proteins synthesized here might be used for the reorganization of synaptic processes which can store the learned information^4–6^. The most interesting gene found here was rho GTPase-activating protein 44-like (*Arhgap44*), which is known to be involved in synaptic plasticity and can promote spine morphological changes associated with long-term potentiation using hippocampal neurons^30–32^.

The 35 downregulated genes in the 4-hour Learning group (Cluster 1 and Cluster 5) were mainly involved in ‘Protein binding’ (GO:0005515), ‘Cell communication’ (GO:0007154), ‘Signalling’ (GO:0023052) and ‘Metabolic process’ (GO:0008152) (Supplementary File S3). Gene heat shock protein 83-like (*Hsp83*) was found in Cluster 5 in our study, and other studies have revealed that *Hsp83* was associated with learning and can response to stimulus^19,20,33^. One potentially interesting gene found here was eukaryotic translation initiation factor 4E-binding protein 2 (*EIF4EBP2*), which is a repressor of translation initiation and has been shown to be involved in synaptic plasticity, learning and memory formation^34,35^.

## Discussion

In this study, we examined dynamic gene expression changes after associative colour learning. Gene expression changes were seen immediately after new colour learning and different sets of genes were up- or down-regulated four hours after training, which may be responsible for long-term memory formation. A limitation of our design is that we are unable to clearly separate the effects of novel colour learning from other sources, and so we are careful not to overstate the significance of any differentially expressed genes in the 4-hour Learning group, other than relative to the 0-hour Learning group. The comparison between the 0-hour Learning and 4-hour Learning allowed us to examine the persistence of the effects of novel colour learning found in the 0-hour Learning group over time and determine potential candidate genes for the underlying mechanisms of long-term memory formation. Given our experimental set-up, we are fairly confident in the observed effects of learning at the 0-hour mark (as compared to the 0-hour no learning group) and their ephemeral nature (as compared to the 4-hour Learning group).

Transcriptomic expression patterns have been shown to be associated with many different aspects of bee behaviour, such as division of labour, foraging experience, social behaviours, and learning and memory formation^36–39^. For instance, thousands of differentially expressed genes were detected when comparing gene expression between bee nurses and foragers^39^; 500 genes were found to be correlated with duration of foraging experience^36^; and previous studies in honeybees have also looked for learning and memory-related genes by comparing bees that have learned and those that have not learned and found 388 (visual learning^33^), 259 (olfactory learning^19^) and 77 (olfactory learning^20^) differentially expressed genes. The number of differentially expressed genes found in our study was also relatively small. All these findings suggest that specific memory formation, compared to the behavioural and physiological transitions from one lifestyle to another, or the many environmental influences that come with foraging experience, involves a moderate number of genes. The number of genes related to visual learning and memory found in this study was much smaller than that found by others^33^. This may be due to the fact that the two time points we tested were closer together and that we controlled for bees’ foraging experience, reward level and the time of collection more strictly, each of which would reduce the number of DEGs.

The upregulated genes in the new colour learning group collected immediately after training (compared with control group) included several genes encoding enzymes, such as phosphatase, methyltransferase, and the synaptic-related genes *Rab10* and *Shank1*. Most of the detected pathways fell under the categories of ‘Signal transduction’ and ‘Nervous system’. In support of our findings, Hoedjes and colleagues have demonstrated the enrichment of signalling-related genes immediately after conditioning in a wasp species that forms long-term memory after only one conditioning trial, such as SLIT-ROBO Rho GTPase-activating protein (*SRGAP1*) and Glutamate receptor subunit 1 (*GluR1*)^40^.

The Rab family of proteins (GTPases) are responsible for vesicle formation, transport and fusion with membranes, and play significant roles in cognitive functions^41^. Rab10 regulates neuropeptide release from vesicles in the nematode *C. elegans*^42^ and is required for dendrite arborisation and axon growth^43,44^. The Shank family of proteins are required for the development and function of neuronal synapses. Shank1 regulates excitatory synaptic strength, promotes dendritic spine maturation and spine head enlargement, and enhances presynaptic function^45,46^. One study reports that Shank1-deficient mice displays enhanced performance in a spatial learning task, while their long-term memory retention in this task is impaired^47^, which suggests an important role of Shank1 in memory formation. It is noteworthy that three genes (*Rab10*, *Hsp83*, LOC100642507 (DNA polymerase beta)) upregulated in the 0-hour Learning group belong to the GO term category ‘Response to stimulus’. The overexpression of these genes may enable organisms to respond properly to environmental stimuli at both the cellular and behavioural levels. The function of some signalling-related genes detected here (e.g. *tws* and *Dusp10*) have not yet been explored in detail and their roles in learning and memory are unclear, which should be examined in future work.

The genes that were upregulated in the 4-hour new colour learning group (compared with the 0-hour new colour learning group) were related to transcription and translation based on GO and KEGG enrichment analysis. It seems that building of new proteins is the predominant activity during the four hours after training. Transcription is necessary for long-term memory formation and at least two transcription waves are required: the first occurs during or shortly after training and the second occurs 3–6 hours after training^6^. Our findings are consistent with previous reports that a small set of immediate-early genes are involved in the first wave and then their protein products trigger the expression of more target genes several hours later, which are responsible for synaptic reorganization through protein synthesis^4–6^.

The genes found here included the upregulated gene *Arhgap44* (rho GTPase-activating protein), the downregulated gene *Hsp83* (heat shock protein 83-like) and *EIF4EBP2* (eukaryotic translation initiation factor 4E-binding protein 2) in the 4-hour Learning group. Rho GTPase-activating proteins, with their substrates Rho-family GTPases, regulate multiple processes in neuronal systems, such as axonal and dendritic growth, remodelling of spines and formation of synapses^30–32,48^. Hoedjes and colleagues also detected the Rho signalling pathway by comparing gene expression in the heads of wasps at three different time points after odour conditioning^40^. The family of heat shock proteins is produced by cells in response to stressful conditions (such as heat shock and exposure to heavy metals) and the upregulation of heat shock proteins protect the cell from impairment caused by these stresses^49,50^. Several transcriptomic studies of bees and wasps show that heat shock proteins are associated with foraging activity^36,37,51,52^, and learning and memory formation^19,20,33^. The high expression of *Hsp83* in 0-hour groups (bees were collected immediately after training) in our study may prepare the bees to respond appropriately in challenging foraging conditions and learning. The translation repressor EIF4EBP2 has been shown to be involved in synaptic plasticity, learning and memory formation^34,35^. The above results suggest that signalling- and protein synthesis-related genes are activated several hours after new colour learning, which may modulate synaptic plasticity. Subsequently, the reorganization of synaptic processes can store the learned information.

In summary, our study shows for the first time the dynamic and temporal transcriptional expression patterns involved in long-term memory formation in bees following visual learning. Bioinformatical analyses showed that the genes triggered immediately by new colour learning were associated with signal transduction; and the genes upregulated four hours after training were related to transcription and translation, which suggests building of new proteins is the predominant activity four hours after training. We also identified the candidate genes (e.g. *Rab10*, *Shank1* and *Arhgap44*) involved in bumblebee colour learning, and the functions of these in learning and memory formation should be explored in future work. Bumblebees have been shown to display a variety of impressive cognitive abilities, such as ball rolling, string pulling, and emotion-like states^53–55^, and the understanding of basic mechanisms of simple memory is necessary before we can utilize this powerful model to unravel the transcriptomic architecture of complex cognition.

## Methods

### Animals

Bumblebee (*Bombus terrestris*) colonies, with around 20 workers, were purchased from Biobest Belgium NV (Westerlo, Belgium). All colonies were housed in wooden nest boxes (40 × 28 × 11 cm), which were connected to small flight arenas (65 × 45 × 25 cm) through a Perspex corridor (25 × 3.5 × 3.5 cm). We manually controlled when individual bees entered the arena. Bee identity was tracked with individual number tags (Opalithplättchen, Warnholz & Bienenvoigt, Ellerau, Germany) glued to the top of the thorax. Bees were marked under red light (epiLED Deep Red 640–660 nm, Futureeden), since red light is in the periphery of their visual spectrum and they can therefore see it only poorly^56^, to ensure visual colour information for bees was kept at a minimum. During experiments, illumination in the lab was controlled with a 12 h day-night cycle (8:00 am−8:00 pm). Bees had no foraging experience until pre-training and all bees used in the experiments had similar age (11–13 days) at collection.

### Behavioural procedures and bee sample collection

#### Pre-training

All bees were first trained to visit transparent Perspex chips (25 × 25 mm) with 7 μl 40% sucrose solution. Five chips were arranged in a pseudorandom array within the arena, each on top of a small transparent glass vial. Only bees that successfully foraged from the transparent chips and returned to the colony 8–10 times with an inter-trip interval under 5 minutes were included in the experiments.

#### Training

On Day 1, bees were trained individually to forage on only one transparent chip, containing 100 µl 40% sucrose solution. Worker bumblebees of similarly large size were selected visually. This was simply to ensure that each bee would consume the entire 100 µl sucrose solution during training trips. After consumption, bees would naturally return to their nest to unload their collected crop load. Each bee had five foraging trips and the inter-trip interval was 10 minutes. Inter-trip intervals were kept consistent because bees always attempted to leave the colony prior to the 10-min time point and we would only need to prevent the bee from entering the arena using small doors in the corridor until the 10-minute interval had ended. Bees tended to return from their nest to the corridor every few minutes and therefore when a bee returned within a minute or two from when the 10-minute interval would end, the bee was prevented from leaving the corridor by closing the corridor doors until it was time for the next trip. Chips were moved to pseudorandom locations in the arena between trips to prevent bees from associating certain spatial locations with reward or colour. On Day 2, the bees received the same training as on Day 1. On Day 3, the bees were divided into three groups (Fig. 1). Bees in the first group received the same training on one transparent chip as Day 2 and were collected immediately after training. The second group received similar training procedures as Day 2, except the transparent chip was replaced by a yellow chip and the bees were collected immediately after training (0-hour Learning). The third group was trained in the same way as the second group, but was left in the hive for four hours without any further foraging experience, and was then collected (4-hour Learning). Bees were collected in liquid nitrogen and stored at –80 °C until dissection. On Day 3, the 4-hour Learning group was trained in the morning (starting at 10am) and the 0-hour Control and 0-hour Learning groups were trained in the afternoon (starting at 2 pm) to make sure all bees were collected at approximately the same time point of the day (2.50 pm). This was to avoid the possibility that any differences in gene expression might simply be due to circadian changes. For each of the three conditions (0-hour Control, 0-hour Learning and 4-hour Learning), 10–12 bees were collected from each of three separate colonies.

#### Retention test

Forty-five bees not used for sequencing were used to validate whether our training procedures actually lead to long-term memory formation. Bees were trained on one yellow chip (or one magenta chip) containing 100 µl 40% sucrose solution, and each bee had five foraging trips with 10 min inter-trip intervals. The retention test, conducted three days after training, required bees to forage among five yellow and five magenta chips each containing 100 µl water. Bees’ landings over three minutes were recorded and the percentage of correct landings was calculated. A landing was defined as any time the bee was positioned on top of a chip and not flying for any amount of time.

### Total RNA extraction, RNA-seq library construction and high-throughput sequencing

Whole brains were dissected out over dry ice and washed in cold phosphate buffer saline (PBS) to remove small pieces of hair or trachea. Ten to twelve bee brains were pooled in each sequencing sample for RNA extraction. Three pooled biological samples were collected for each of the three conditions (0-hour Control, 0-hour Learning, 4-hour Learning). Total RNA was extracted from whole brains using Trizol reagent (Invitrogen, Carlsbad, CA, USA) according to the manufacturer’s instructions. RNA quantity and integrity were measured using a 2100 Bioanalyzer (Agilent). The RNA concentration and the RNA integrity number (RIN) are shown in Supplementary Table S1. RNA-seq libraries were generated using NEBNext^®^ Ultra™ RNA Library Prep Kit for Illumina (New England Biolabs Inc., Ipswich, MA, USA). Qualification and quantification of the libraries were conducted by Agilent 2100 Bioanalyzer and ABI StepOnePlus Real-Time PCR System separately. Finally, the libraries were paired-end sequenced using Illumina HiSeqTM 2000, which generates around 50 million paired-end 150 bp raw reads for each sample. Library construction and sequencing were conducted by BGI Genomics Co., Ltd. (Shenzhen, China).

### Read mapping and gene expression calculation

Primary sequencing data produced by Illumina HiSeqTM 2000 were raw reads. Before data analysis, the reads with adapters, reads with more than 10% unknown bases and low quality reads were removed from raw reads. Quality control was performed on the remaining reads by drawing a base composition chart and a quality distribution chart, to ensure that each sample possesses balanced base composition and high sequencing quality.

The cleaned reads were then aligned to the *Bombus terrestris* genome and reference genes (http://www.ncbi.nlm.nih.gov/genome/2739?genome_assembly_id=34093). Burrows-Wheeler Aligner (BWA)^57^ was used to map cleaned reads to the reference genome and Bowtie^58^ was used to reference genes. The mapping ratio and the distribution of reads on bumblebee reference genes were calculated to evaluate the sequencing quality. Gene expression levels were quantified using RNA-Seq by Expectation Maximization (RSEM) software^59^. Then the fragment counts were normalized to Fragments Per Kilobase of transcript per Million mapped reads (FPKM), which eliminated the influence of different gene length and discrepancy of the library size. FPKM values were used for gene expression analysis.

### Identification of differentially-expressed genes and cluster analysis

One-way ANOVA was used to identify differentially expressed genes (DEGs) among the three experimental groups. Genes were considered differentially expressed with a p-value <0.01 and |Fold change| ≥ 2. Principal component analysis (PCA) was performed on normalized FPKM values of the DEGs to detect global gene expression patterns in each sample. To find genes with similar expression patterns, hierarchical clustering analysis was conducted on the normalized FPKM values of DEGs. All statistical analysis was conducted with MATLAB 9.2 (MathWorks, Natick, MA, USA).

### Gene ontology and pathway enrichment analysis of DEGs

Functional analysis of DEGs was obtained by performing gene ontology (GO) and KEGG pathway enrichment analysis, which were conducted using a strict algorithm developed by BGI Genomics Co., Ltd. (Shenzhen, China) (see details^19^). The p-value was corrected through Bonferroni Correction^60^ and the corrected p-value threshold of 0.05 was used to detect significantly enriched GO terms and pathways. WEGO software^61^ was used to do GO functional classification for DEGs to determine the distribution of gene functions.

### Data availability

The datasets generated or analysed during the current study are available from the corresponding author on reasonable request.

## Electronic supplementary material

Supplementary Information

Dataset 1

Dataset 2

Dataset 3

Dataset 4
