# Supplementary material for: Large-scale transcriptome changes in the process of long-term visual memory formation in the bumblebee, Bombus terrestris
Source: Sci Rep. 2018 Jan 11;8:534. doi: 10.1038/s41598-017-18836-3 (PMC5765018; doi:10.1038/s41598-017-18836-3)
Supplement: Supplementary file 1 — Supplementary Information [file 41598_2017_18836_MOESM1_ESM.pdf]

Title: **Large-scale transcriptome changes in the process of long-term visual memory formation in the bumblebee, *Bombus terrestris***

Li Li[1]*, Songkun Su[2]*, Clint J. Perry[1], Maurice R. Elphick[1], Lars Chittka[1], Eirik Søvik[3]

# Supplementary Information

## Supplementary Figures

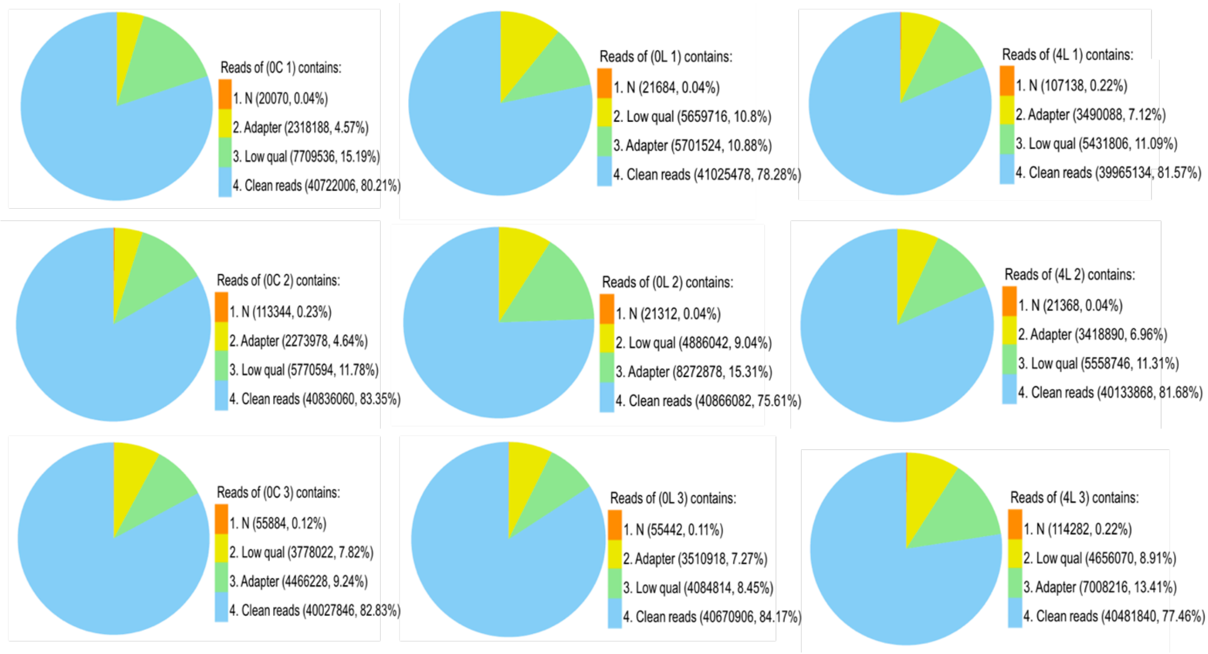


**Supplementary Figure 1 | Classification of raw reads for each sample.** Before data analysis, the reads with more than 10% unknown nucleotides (N), with adapters (Adapter), and low quality reads (Low qual) were removed from raw reads. The remaining reads were the clean reads. The value indicates reads number and its ratio on raw reads. 0C: 0-hour Control; 0L: 0-hour Learning; 4L: 4-hour Learning.

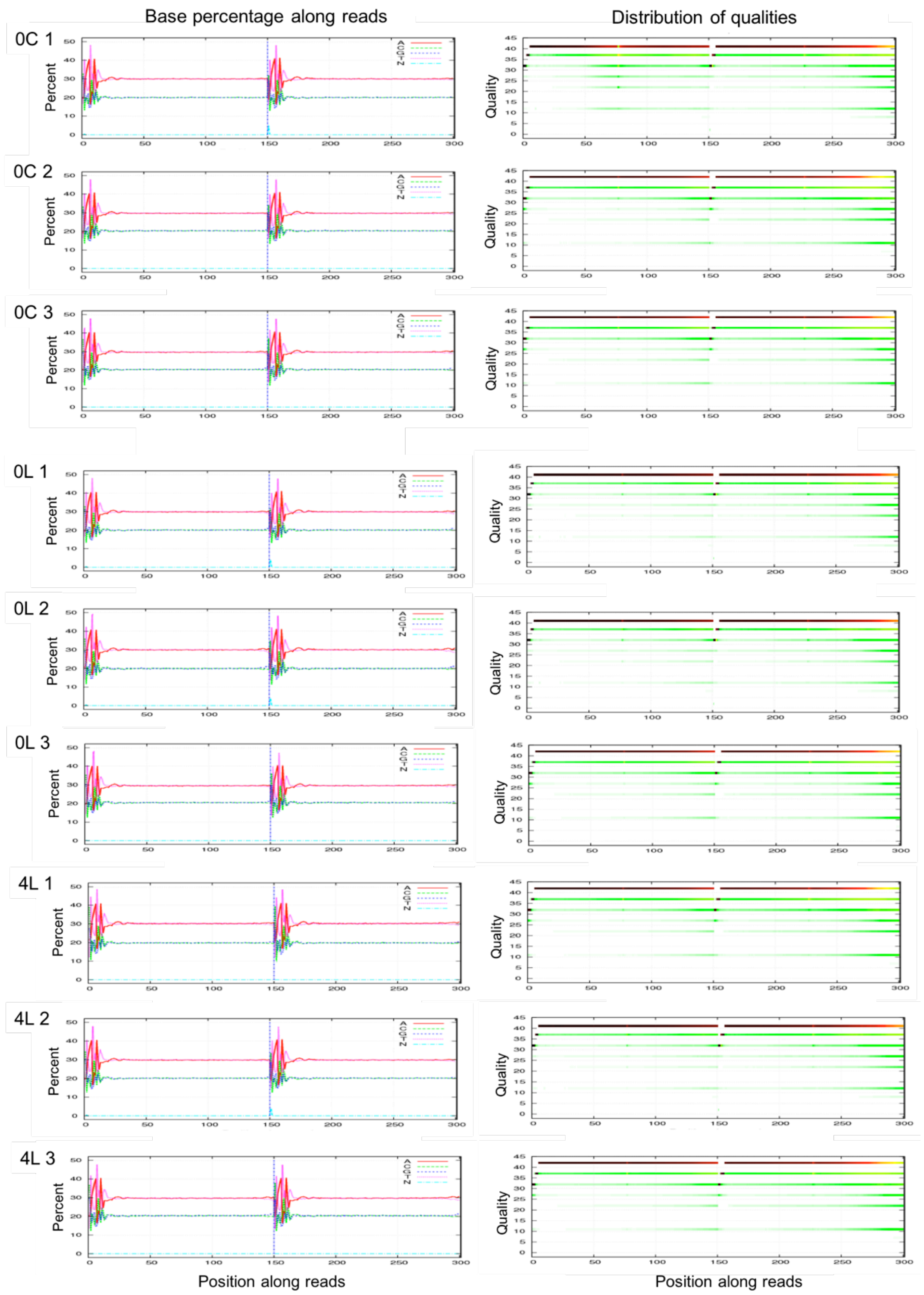


**Supplementary Figure 2 | Quality assessment of sequencing paired-end reads. (Left) The nucleotide composition of RNA-Seq reads.** The percentage of each nucleotide (Y-axis) is plotted against read length (Y-axis; 1-150bp and 151-300bp represent the reads from end 1 and end 2 separately). Colors indicate different nucleotides (A = red; C = Green; G = Blue; T = Magenta; N: Light Blue). A curve should be overlapped with T curve, while G curve be overlapped with C curve, which can be seen from all our

samples. If abnormal condition happens during sequencing, it may show an unbalanced composition. Note that the changing composition in the beginning of reads is common in RNA-Seq data. (**Right**) **Quality distribution of bases along RNA-Seq reads.** Each base quality (Y-axis) is plotted against read length (X-axis; 1-150bp and 151-300bp represent the reads from end 1 and end 2 separately). Quality score reflects the sequencing error rate and the relationship between them is sequencing error rate 1%, 0.1% and 0.01% corresponds to quality score 20, 30 and 40. Most of base positions in our sequencing showed good quality (score >20). 0C: 0-hour Control; 0L: 0-hour Learning; 4L: 4-hour Learning.

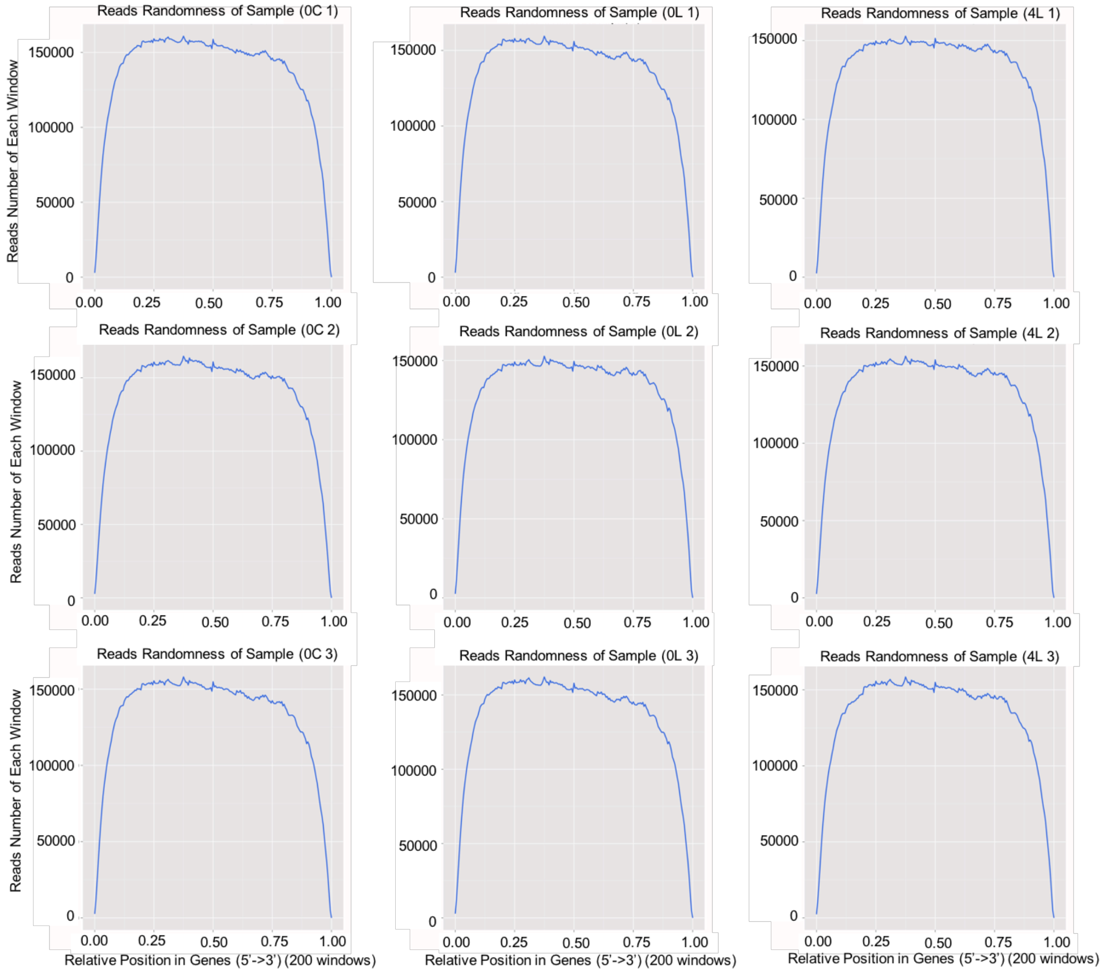


**Supplementary Figure 3 | The distributions of reads on bumblebee (*Bombus terrestris*) reference genes.** X-axis is the relative position in genes which is calculated as the ratio between read location and the gene length and Y-axis is the number of reads. Reads should be evenly distributed on reference genes, otherwise it means the randomness is poor (i.e. reads prefer to specific gene region) which will affect

following analysis. The read randomness in all our samples is good as shown in the figure. 0C: 0-hour Control; 0L: 0-hour Learning; 4L: 4-hour Learning.

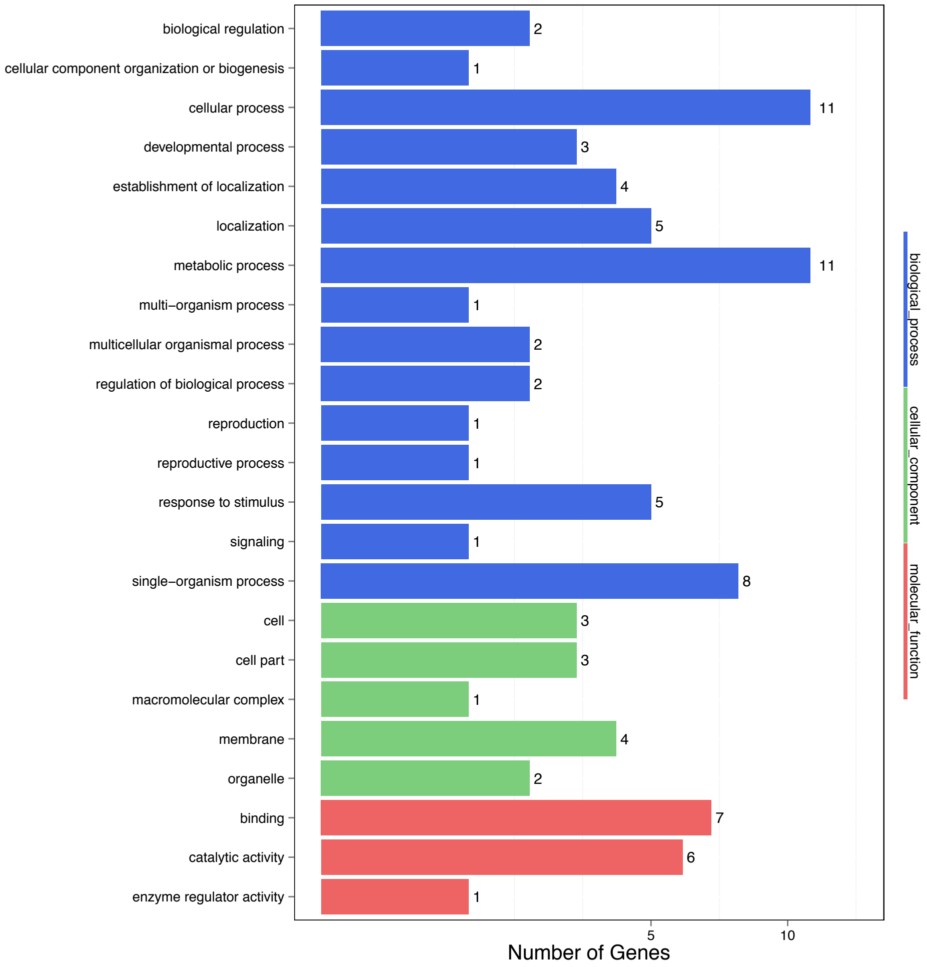


**Supplementary Figure 4 | GO functional classification of differentially expressed genes (DEGs).** The DEGs were annotated into three main categories: biological process, cellular component and molecular function. The number of genes in each GO terms were displayed and 58 of the 110 DEGs belonged to biological process.

## Supplementary Table

**Supplementary Table 1 | The quality of RNA samples used for sequencing.** RNA concentration and integrity were measured by Agilent 2100. Level A means the sample is qualified and the amount of sample satisfies two times library construction or more. 0C: 0-hour Control; 0L: 0-hour Learning; 4L: 4-hour Learning.

| Sample Name | Concentration (ng/μl) | Volume (μl) | Total Mass (μg) | RIN | Library Type | Test Result |
|---|---|---|---|---|---|---|
| 0C 1 | 456 | 55 | 25.08 | 7.6 | HiSeq Eukaryotic Transcriptome | Level A |
| 0L 1 | 474 | 55 | 26.07 | 7.7 | HiSeq Eukaryotic Transcriptome | Level A |
| 4L 1 | 393 | 56 | 22.01 | 7.9 | HiSeq Eukaryotic Transcriptome | Level A |
| 0C 2 | 480 | 57 | 27.36 | 7.9 | HiSeq Eukaryotic Transcriptome | Level A |
| 0L 2 | 516 | 57 | 29.41 | 8 | HiSeq Eukaryotic Transcriptome | Level A |
| 4L 2 | 627 | 57 | 35.74 | 7.9 | HiSeq Eukaryotic Transcriptome | Level A |
| 0C 3 | 528 | 57 | 30.1 | 8 | HiSeq Eukaryotic Transcriptome | Level A |
| 0L 3 | 549 | 54 | 29.65 | 8 | HiSeq Eukaryotic Transcriptome | Level A |
| 4L 3 | 639 | 57 | 36.42 | 7.9 | HiSeq Eukaryotic Transcriptome | Level A |
